# Steganography in IoT: Information Hiding with Joystick and Touch Sensors

**DOI:** 10.3390/s23063288

**Published:** 2023-03-20

**Authors:** Katarzyna Koptyra, Marek R. Ogiela

**Affiliations:** Cryptography and Cognitive Informatics Laboratory, AGH University of Science and Technology, 30-059 Kraków, Poland

**Keywords:** steganography, IoT, sensor, touch, joystick

## Abstract

This paper describes a multi-secret steganographic system for the Internet-of-Things. It uses two user-friendly sensors for data input: thumb joystick and touch sensor. These devices are not only easy to use, but also allow hidden data entry. The system conceals multiple messages into the same container, but with different algorithms. The embedding is realized with two methods of video steganography that work on mp4 files, namely, videostego and metastego. These methods were chosen because of their low complexity so that they may operate smoothly in environments with limited resources. It is possible to replace the suggested sensors with others that offer similar functionality.

## 1. Introduction

Steganography is a technique of covert communication that involves hiding sensitive information in an ordinary-looking message. The other names of steganography are “secret writing” or “hiding in plain sight”. Its  main goal is to prevent detection, therefore, the secret data are most often embedded within a file of a common type. Steganographic algorithms operate on multiple media, for example, texts [1,2], images [3,4,5,6], videos [7,8], network packets [9,10,11], binary executables [12] and many more [13,14,15,16]. Besides remaining above suspicion, good carriers should also offer reasonable capacity. A branch of steganography which conceals more than one message in a single container is called multi-secret steganography [17]. Usually, it uses separate embedding algorithms to place data in different parts of the container [18]. Then the messages may be extracted independently.

The most popular carriers for steganography are those with large embedding space, i.e., images and videos. The common aspect of such methods is that they may operate in a spatial domain, frequency domain or that they may encode data in the file structure. In the spatial domain, the best-known algorithm is the least significant bit (LSB), which substitutes some pixel bits for message bits. There are multiple variants of this technique, including different numbers of bits (for example [19,20] substitute four least significant bits) and mapping strategies. Another LSB-based method manipulates bit planes with a binary operator [21] to hide a message. Sometimes, the least significant bit is combined with other ideas, for instance with pixel value differentiation [22] in the technique called five-pair pixel differentiation. Further, a popular approach is to use LSB method together with cryptography. The most common applications in steganography are encryption of the message, addition of the checksum and introduction of randomization to select modified pixel locations [23].

In frequency domain, the most popular approaches of data hiding are with discrete cosine transform [24] and discrete wavelet transform [25]. Some other strategies of concealing a secret message are based on singular value decomposition. These techniques use as embedding region singular vectors, singular values or combinations of them [26,27,28,29]. There are also steganographic methods that use principal component analysis to facial images, which is called eigenfaces [30]. These algorithms generally include encryption for additional security, the examples may be found among image [31] and video steganography [32].

Another approach to steganography is to generate the container from scratch based on the secret message—then an input medium is not required [33]. The most frequent application of this technique is to create fractal images [34] or texts suggestive of a spam [35]. Generation methods have large capacity, as the user is able to create a carrier of any length. Some steganographic papers are focused on the key generation process [36], others combine steganography with other branches of security, for example, [37] discusses application of personal biometric characteristics to data hiding, and [38] presents a method of encryption and decryption of images. More techniques may be found in general surveys that describe the state-of-the-art of steganography [39,40,41,42].

**Figure 1:**
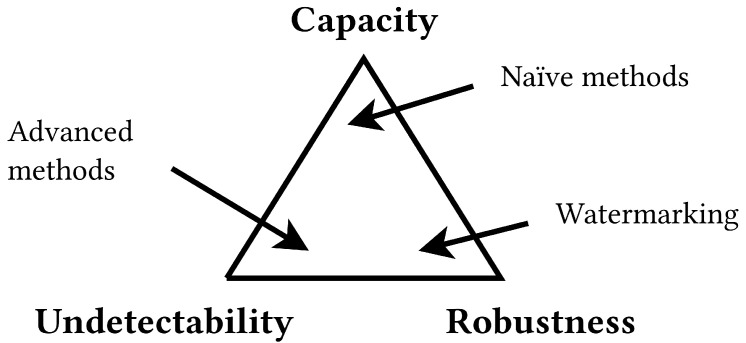
Relationship between steganography requirements.

Every steganographic system is characterized by three parameters: capacity, undetectability and robustness. Capacity shows how much data may be hidden in a carrier. For some methods, it is possible to precisely compute capacity (like 25% of the container), for others, it depends on some features of the medium, like noise level, existence of patterns etc. Undetectability is the most important aspect for information hiding, because when the message is revealed by an attacker, the whole system is compromised. There are two levels of the undetectability: sensational (using sight, hearing etc.) and statistical (finding anomalies by computation). Robustness means invulnerability to modifications, for instance compression, conversion to another format or partial damage. Algorithms with high robustness may save hidden data when an adversary tries to attack the system by performing some operations on the medium. All these features are competing [43] and their importance vary depending on the application, as presented in Figure 1. When high capacity is needed, larger part of the carrier is used for secret data storage. Then more data may be transferred, but at the same time more distortions are introduced to the carrier file. On the other hand, robustness is crucial in watermarking. Such methods usually embed multiple copies of the mark and hence increase changes of defending the message from attacks, but this also reduces undetectability, because it is easier to reveal redundant data.

In the Internet-of-Things, steganography may be used not only to send secret messages, but also to add an additional layer of security to transferred data [44]. The latter application results from lack of security and privacy protection in many IoT systems, as stated in the Open Web Application Security Project [45] which aims to identify top ten critical risks. The authors indicate weak, guessable or hardcoded passwords as among the urgent things that need to be repaired. Among the other serious threats, we may find a lack of a secure update mechanism, insufficient privacy protection, insecure data transfer and storage, etc. Because numerous IoT devices work with digital cameras, good choices of carriers are images and video files. They offer large capacity and do not raise suspicions as their presence is common or even expected in systems equipped with a camera. For this reason, MP4 files have been chosen as carriers. Secret data are embedded with two algorithms, which are videostego and metastego.

In the presented system, there are two user-friendly sensors that provide independent sources of data. Generally, sensors may be divided into input, output and bidirectional. Input devices collect data from the environment. They may measure temperature [46], medical parameters [47], displacement [48], pH [49], pressure [50], humidity [51], inertia [52], etc. Output devices broadcast messages to the external world. These may be LEDs, buzzers, lasers, displays [53] etc. Bidirectional sensors are more complicated modules that allow both input and output of data. In a steganographic system input devices may be used to provide data to be hidden or to manually trigger an event. On the other side, output devices may be applied to signal state of the system, for example being ready to read data, or to indicate unexpected events, like failure of an operation. Sensors chosen to this study serve to input messages directly by the user.

The main goal of this paper is to propose a multi-secret steganography system characterized by following features: sensors allow to input data without attracting too much attention, the embedding algorithms are efficient to work in an IoT environment with limited resources, and data input is easy for the operator.

## 2. Materials and Methods

### 2.1. Hardware

The hardware setup is consisted of input sensors and a platform for gathering data.

#### 2.1.1. Platform

**Figure 2:**
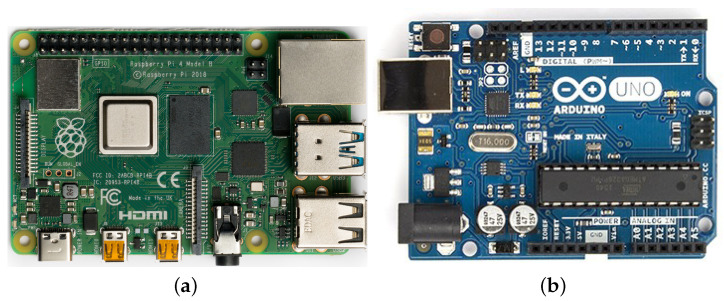
Possible platforms for multi-secret steganography system: (**a**) Raspberry Pi 4 Model B (Laserlicht/Wikimedia Commons/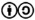); (**b**) Arduino Uno (R.hampl/Wikimedia Commons/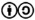).

The project may be based on Arduino and/or Raspberry Pi. Both platforms are built on a principle of open design (Figure 2), but there are some differences among them. For example, only Arduino is able to sense analog inputs. On the other side, Raspberry Pi has an operating system and more processing power. It is possible to establish a communication between mentioned boards using UART interface or wirelessly.

Citing [54], Raspberry Pi 4 Model B is a tiny, credit-card-sized computer, usually used as a robot brain, smart home hub, media center, factory controller, etc. The chosen version has 8 GB of RAM. It is equipped with a 1.5 GHz 64-bit quad core ARM Cortex-A72 processor, two micro HDMI ports, two USB 3.0 ports, two USB 2.0 ports, 802.11ac Wi-Fi, Bluetooth 5 and gigabit Ethernet. The board is powered via a USB-C port and requires a 5-V supply. The default operating system is Raspberry Pi OS (formerly called Raspbian), a Debian-based Linux distribution, but the board may run many other systems.

Arduino platform is based on ATmega328P microcontroller. It is equipped with 14 digital and six analog input/output pins and offers numerous compatible devices and expansion shields. Arduino Uno is a little smaller than Raspberry Pi 4 and may be powered by a USB cable or by an external battery. Additionally, there is specialized IDE cut out for programming Arduino that supports C and C++ (alternatively the board may be programmed via command line interface). The Arduino program consists of two functions—setup that runs once at the beginning and loop for operations performed indefinitely.

Although analog inputs cannot be read by general purpose pins of Raspberry Pi, there is a workaround. One needs an analog-digital converter like the MCP3008. This chip allows to read up to eight 10-bit analog inputs with a single query. Other easy solution is to use Arduino to read analog inputs and then send the data to Raspberry Pi. The boards are connected by a USB cable and the communication is done via the serial port.

When MCP3008 is used, the user should activate Serial Peripheral Interface in Raspberry Pi configuration (raspi-config in command line). Further, camera interface should be enabled to record videos.

#### 2.1.2. Thumb Joystick

**Figure 3:**
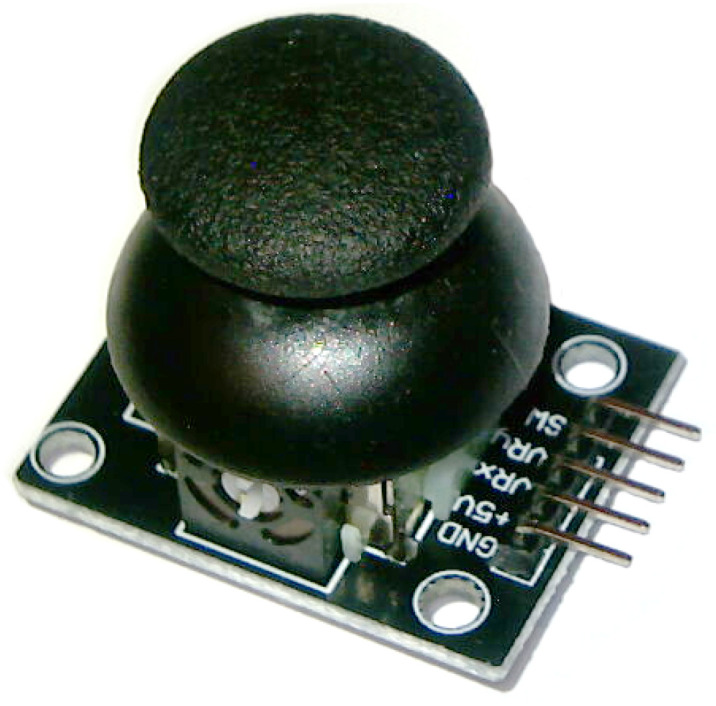
Thumb joystick with button.

Thumb joysticks are designed to measure movements in *x* and *y* axis. They are commonly found in PlayStation2 controllers. The device used in this project has additional button activated by pressing the joystick down. There are five pins: GND (ground), +5V (power), VRx (*x* axis measurement), VRy (*y* axis measurement), and SW (button signal), as shown in Figure 3. Sensing of a movement is realized with two potentiometers, one for each axis. The values measured on VRx and VRy pins are analog and vary from 0 to 1023. SW digital signal is by default LOW, but changes its state to HIGH when the button is pressed. The control with joystick is convenient, as the device provides two degrees of freedom.

#### 2.1.3. Touch Sensor

**Figure 4:**
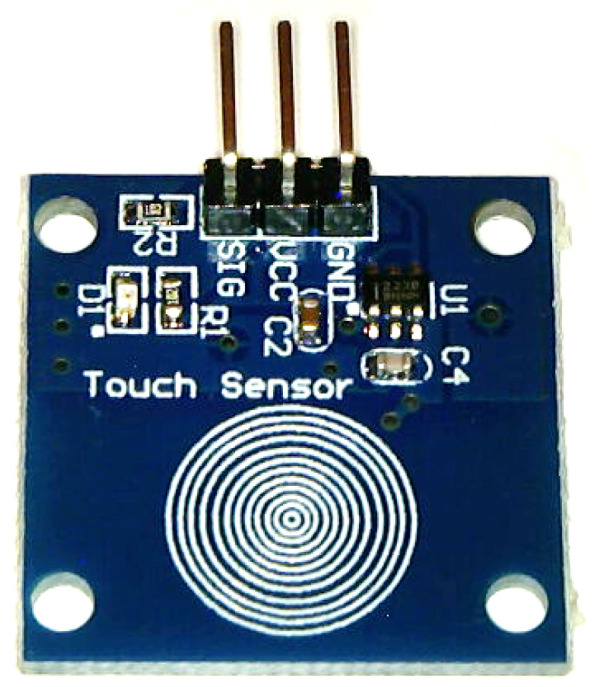
Capacitive touch sensor.

Touch sensor is integrated into a module with three pins, as depicted in Figure 4. GND (ground) pin should be connected to ground of the board, VCC (voltage common collector) to power supply, and SIG (signal) to selected general purpose pin for collecting data. Presented capacitive touch sensor works with a range of currents, including both 5 V and 3.3 V. On the input pin we may receive LOW (by default) or HIGH (if a touch is detected). The response changes when user’s skin makes direct contact with circuit wires. Both sides (positive and negative) of the device are sensitive and may be touched even when the surface of the sensor is covered with a thin paper. For this reason, it is very good for steganographic purposes.

### 2.2. Software

Software reaches two steganographic schemes, each of them consists of embedding and extracting algorithms. These two schemes will be illustrated in the next sub-sections.

#### 2.2.1. Videostego

In a nutshell, videostego is a tool designed to write and read hidden messages in MP4 files using steganography technique of the least significant bit. This method may not be widely known because it was originally published in Spanish [55], so it may be a good idea to present basic assumptions of this stegosystem.

Let’s start with the MP4 format. It defines how to store video, audio and other related information in a single file. One of the most brilliant features of MP4 is that the audiovisual content can be divided to smaller pieces. In this way the format found applications in streaming services, in which there is no need to download a full file at once, but only currently played fragments. The structure of MP4 files is hierarchical [56], and a single unit of data is called block or box. The standard defines which blocks may contain others and where they may occur. At the root level, we commonly encounter file type (ftyp), movie (moov), media data (mdat) and so on; at deeper levels there may be movie header (mvhd), track (trak), track header (tkhd) etc.

The structure of a single block is also specified. The first 8 bytes are reserved for a header, which itself is consisted of two parts: four bytes store size in big-endian and the next four bytes indicate block type. For example header 000000086d646174 means that this blocks is of type mdat and its length is 8 bytes (as can be seen, empty boxes are allowed). If further bytes are present, they depend on the block type.

**Figure 5:**
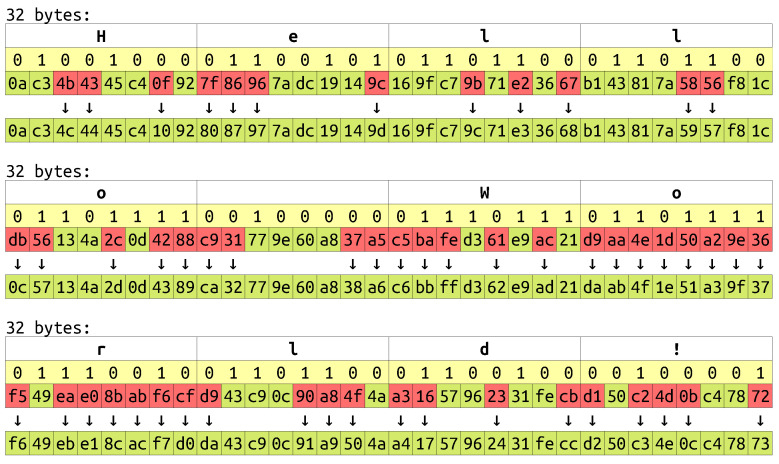
Example of videostego embedding. The hidden message is “Hello world!” (white boxes). Yellow boxes show message bits, red boxes indicate required bit flipping, and green boxes depict resulting bytes.

Returning to videostego method, the embedding algorithm belongs to container modification/substitution family, which means that it changes existing content of a carrier instead of adding new data. Therefore the file size remains the same after embedding. Secret data are hidden in the middle one-third of mdat block. This is where the audiovideo content is stored, so these data may be modified in an unnoticeable way. The middle part is chosen to avoid damaging metadata [55]. Two initial bytes are used to directly store message size (they are replaced). Later the string “vstg” (the signature) is concatenated with the message and the resulting data are concealed. The encoding is realized by flipping the least significant bits of those bytes that do not correspond to the input data. This process is depicted in Figure 5.

Extracting the message requires localization of the proper mdat block, which is the one longer than 8 bytes. Then, to recover the message length, the algorithm reads two bytes from the middle one-third of the block and convert them to integer. Later the secret message is reconstructed from least significant bits of following 8× length bytes, starting from offset 34 (2 bytes of size and 8×4=32 of the signature).

Videostego is able to conceal up to 65,501 bytes, providing that the block size has sufficient length. When the secret message is longer, the algorithm may be modified to use more than two bytes as a size indicator. The implementation is quite simple and the complexity is low. On the other side, files generated by videostego are vulnerable to processing. So the message will probably be destroyed when the video is uploaded to Twitter, LinkedIn or Youtube. In presented IoT system, the video will not be published on such platforms, so this drawback is not very significant.

#### 2.2.2. Metastego

**Figure 6:**
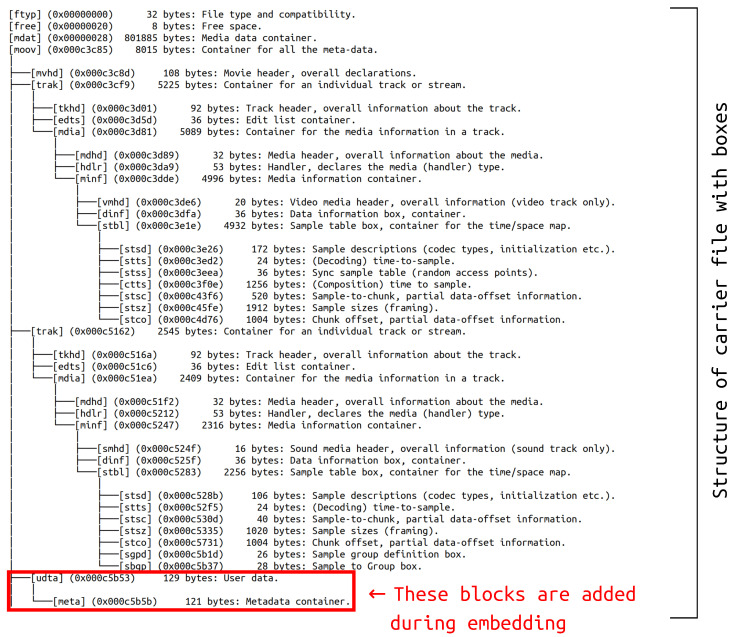
Example of metastego embedding.

Metastego belongs to container modification/injection methods, which means that secret data are introduced into a carrier, but do not replace existing content. In consequence, resulting file is a little bigger than the original. The idea behind this technique is to hide the message in metadata. It is realized by injecting user data (udta) block with metadata (meta) box inside. The meta block has its own structure, but from steganographic point of view the most interesting is comment (©cmt). Such tag may contain up to 255 bytes of UTF-8 data. This is where the message is placed. The architecture of exemplary file after metastego embedding is showed in Figure 6. The red rectangle indicates blocks with hidden data. Additionally, the picture demonstrates tree structure of this mp4 file in which some boxes are located inside others.

Before embedding, the message is encrypted—stored with a keystream and encoded in base85. This step is needed to avoid detection by a simple examination of the file content. Base85 is less popular coding method than base64, but has more non-alphanumeric characters and better compression. It has been chosen because resulting data is not as characteristic as in base64, so hidden data are more difficult to spot and recognize. Later the comment is created and placed in an appropriate position of the carrier. It should be noted that some other parts of the file are also modified, for example the superior block size. To extract data, the algorithm reads the content of the comment, decodes it and performs xor operation on the recovered ciphertext and the key.

Presented method is fast and may be applied in the IoT environment. As can be seen, videostego and metastego use different areas of container to embed secret data. Thanks to that the messages do not collide and both may be recovered in a lossless way.

**Figure 7:**
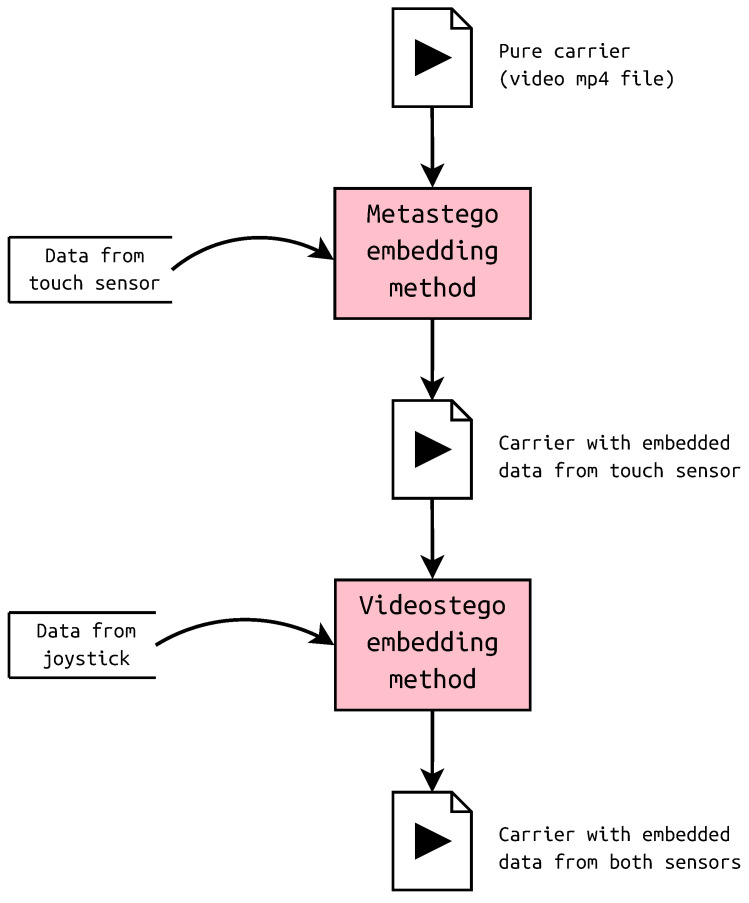
Embedding order.

Videostego has been used for hiding data from the joystick, while metastego—from the touch sensor. Both messages were encoded in the same container in the following order: first metadata, than least significant bits, as presented in Figure 7. In fact, the sequence of operations is a matter of implementation, the reverse order is also possible.

**Figure 8:**
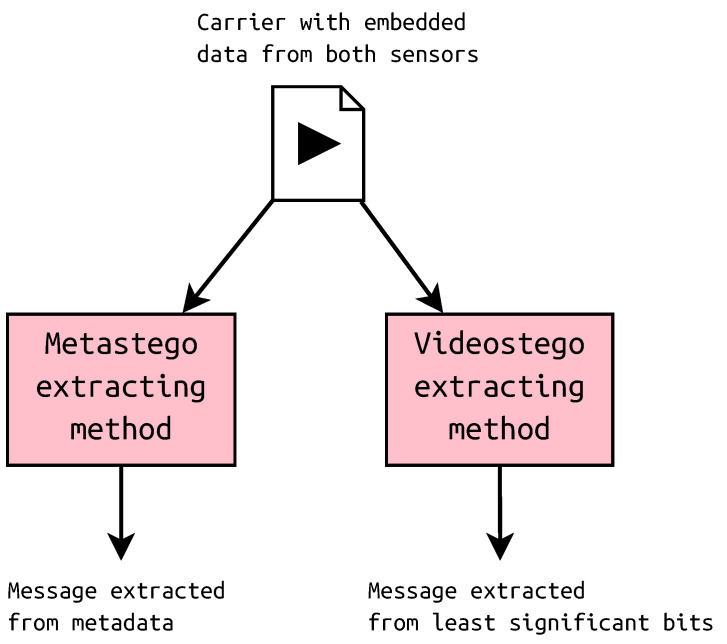
Extracting the hidden messages.

The extracting may be done in any order as the messages are placed in other regions of the container. It is possible to recover only one message if necessary. Both extracting algorithms take the same carrier as an input, but they return other data, as depicted in Figure 8.

## 3. Results

**Figure 9:**
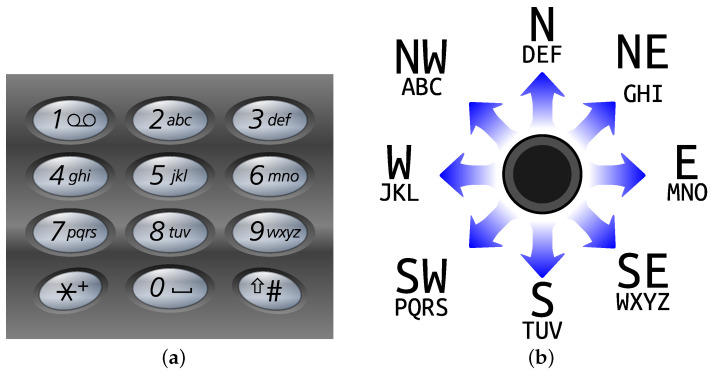
Hiding data with a joystick: (**a**) Keypad of an old telephone; (**b**) Mapping from joystick positions to letters.

**Figure 10:**
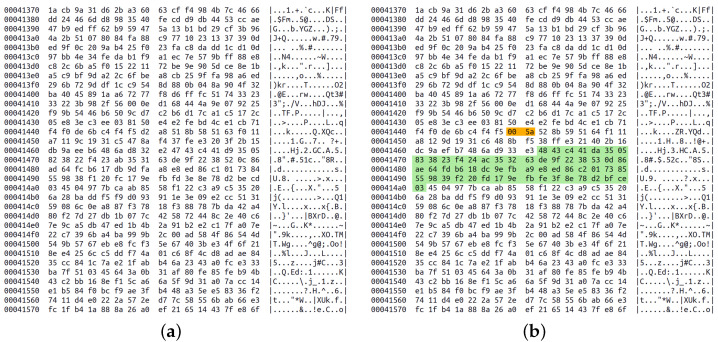
Videostego method: (**a**) carrier before embedding; (**b**) carrier after embedding, two orange bytes encode the length, green bytes contain secret data in their least significant bits.

Signal from the joystick is a pair of numbers (the additional button has not been used) which needed to be translated to a meaningful communicate. The solution has been inspired by old phones with a keypad (Figure 9a). The domain has been divided into nine areas according to directions, i.e., N, S, W, E, NW, NE, SW, SE and neutral (Figure 9b). Each area has assigned letters that may be entered by moving the joystick back and forth in a specific direction. For instance, to choose letter K, the user should quickly change joystick position to W, then neutral, then again W and finish in neutral position. The message created in this way has been concealed with videostego method. In presented example we wanted to hide “WELCOME”, so the entered signal was: SE; N, N; W, W, W; NW, NW, NW; E, E, E; E; N, N. Figure 10 shows differences caused by the embedding process. It may be seen that introduced modifications are very subtle and are not visible when the video is played. Additionally, the file size did not change. Recovering the secret is done by finding the region in which the message had been hidden and reading the least significant bits of those bytes. In this case, we obtained 01010111010001010100110001000011010011110100110101000101 which is translated to “WELCOME” in ASCII. The test for the modified version of the videostego algorithm (without vstg signature) gave very similar results, so it is omitted.

**Figure 11:**
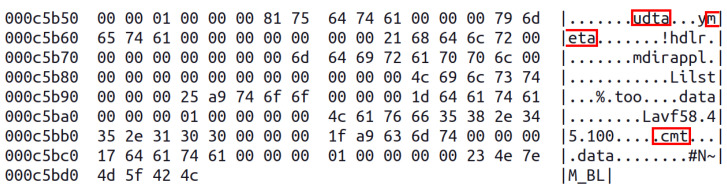
Result of embedding secret in metadata.

On the other hand, the touch sensor is only able to detect two states: touched or not. The user may, however, control the duration and the frequency of each signal. For this reason the data are introduced in Morse code, for example ····∣·∣·−··∣·−··∣−−−. Figure 11 shows final part of the carrier with visible metadata and indicated important parts. At the end there is a message.

The extracted ciphertext is #N∼M_BL, decoding from base85 gives c4e5792b23 in hex. It is then xored with the keystream 8ca035676c and in result we obtain 48454c4c4f, which is “HELLO” in ASCII. The contents of the video remained untouched, the changes are only present in user data. The file size increased by 31 bytes which is a negligible fraction of its original size.

The sensors effectiveness in data input was checked by measuring the number of gestures needed to introduce a selected word. For example, to write letter K with the joystick, we need two left gestures. On the other hand, three gestures are required with the touch sensor (−·−) because both dash and dot are counted as a single gesture. The tests were conducted on the Longman Communication 3000—“a list of the 3000 most frequent words in both spoken and written English, based on statistical analysis of the 390 million words”. These words cover most of the language and allow to understand at least 86% of the content [57]. Each position on the list is marked with symbols: “W1, W2, and W3 for words that are in the top 1000, 2000 and 3000 most frequent words in written English, and S1, S2 and S3 for the top 1000, 2000 and 3000 most frequent words in spoken English”. These categories do not necessarily overlap, which means that a word may be higher in one rank than in other, or even be present in only one category. For example, “hello” is marked S1, in other words is one of the top 1000 words of spoken English, but does not belong to the top 3000 most frequent words in written English.

**Figure 12:**
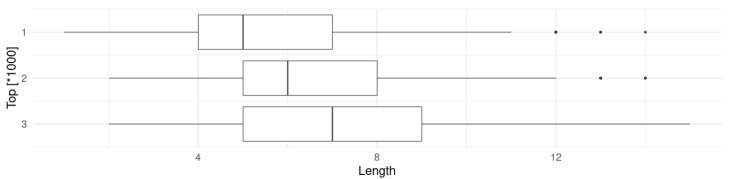
Graphical summary of lengths of most frequent words in English.

For tests, three categories have been created: Top1000 for 1000 most frequent words (both spoken and written), Top2000 and Top3000. Top1000 is a subset of remaining categories, but it is intentional to see how the wordbase changes when new entries are added. Table 1 presents summary of word lengths for each category. It is clearly visible that more frequent words are shorter on average. The database contains lowercase-only entries, therefore a small preprocessing has been applied: changing all uppercase letters to lowercase (for example OK, TV) and remove non-alpha characters (for example so-called). The same data are also presented in Figure 12.

**Figure 13:**
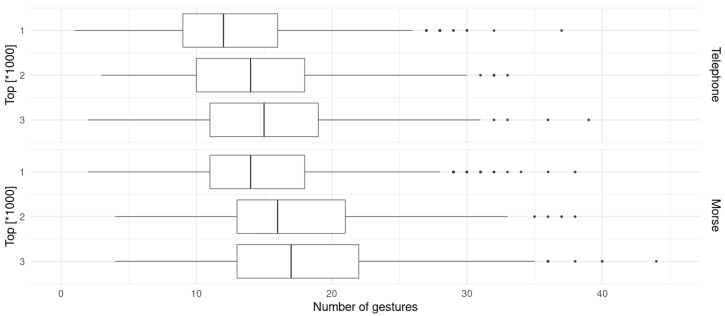
Graphical summary of number of gestures needed for telephone-encoded words (**top**) and morse-encoded words (**bottom**).

A required number of gestures for both kinds of encoding was calculated for every word in the Longman list. Considering the joystick, a back and forth movement is counted as a single gesture. For the touch sensor, a single gesture is either dot · or dash −. The summary of exact values is presented in Table 2 and the graphical representation (boxplots with the median, two hinges and two whiskers) is in Figure 13. The graphs have identical limits of *x* axis to make them easy to compare.

According to the data, the number of gestures in roughly similar, but on average a little longer in morse-encoding. As a remainder, telephone-encoding was used with the joystick, and morse-encoding with the touch sensor. This means that the joystick is characterized by greater efficiency by about 13–14%.

Last but not least, the user experience with the sensors depends on the initial skills of the operator. People familiar with keyboard phones require almost no training for data entry with the joystick. They can introduce messages fast and smoothly. Considering the touch sensor, users without knowledge of morse code need a cheatsheet and then data entry is noticeably slower than trained people.

## 4. Discussion

Let us recall the three main assumptions of the presented project. They are ease of handling, inconspicuous data entry and low complexity. The first two goals are hardware-related and the third is software-related. Below are described their characteristics, possible implications and comparison to other solutions.

**Figure 14:**
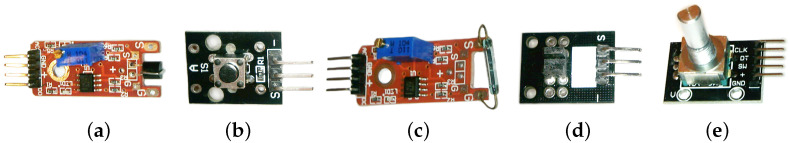
Other tested sensors: (**a**) another touch sensor; (**b**) button; (**c**) reed switch; (**d**) photo interrupt sensor; (**e**) rotary encoder.

For example, another steganographic system [54] uses APDS-9960 proximity and gestures sensor. Secret data are concealed within a comment section of time-lapse photographs (JPEG files) captured with a digital camera on Raspberry Pi. The sensor may operate in two modes: to directly enter data, or to trigger an event. That architecture with multi-purpose device is quite different than presented in this paper. Here, two simple sensors provide sources of data for distinct embedding algorithms. Secrets are hidden inside a single carrier (mp4 video), but in separate sections. The linking aspect of these systems is similar IoT platform. Because sensing devices are rarely incorporated in steganographic research, the evaluation of their usefulness is a bit challenging. Therefore, several sensors have been tested besides of the suggested two. Some of them are presented in Figure 14. Another touch sensor from Figure 14a functions almost exactly the same as the chosen one, but has lesser operating area and turned out to be a little less comfortable. The contact surface of the winning sensor is considerably big, so the user does not have to be overly accurate in introducing data. The button from Figure 14b requires more strength to be activated as it must be pressed, not touched. Therefore data entry is longer than in touch sensor. Additionally, a noticeable click is heard during operation, so the button has been disqualified for steganographic applications. A better solution is reed switch module from Figure 14c. It detects magnetic field by closing the circuit when the magnet is nearby. Data entry is quite fast, but it is difficult to use this sensor inconspicuously. It may be done, for example, with a ring with embedded magnet, so from practical point of view it is worse than the touch sensor. Similar note goes to photo interrupt sensor from Figure 14d. The user may introduce data by placing an object (like a piece of paper) between both sides of the device which blocks the light. The speed is comparable with the button, but using this sensor without drawing attention calls for creative thinking. The last example is a rotary encoder from Figure 14e. It encodes data by left or right rotation. One of the possible solutions for message entry is to make a binary tree with alphabet letters in its leaves and to choose left or right path in each step. This is much slower and less convenient than the joystick because in rotary encoder entering, one letter needs five gestures, while in a joystick, there are usually three and sometimes four.

There are not many sensors that offer multiple degrees of freedom. One of such devices is a gyroscope. However, it is very hard to create an usable system of data entry which is not burdensome for the user. For this reason, the gyroscope has been considered as inadequate for set goals. On the other hand, the joystick turned out to be very easy to learn and after a few minutes of training, messages could be introduced correctly. Rejected sensors may be very good for other applications, but for the presented project the winners are the touch sensor and the joystick. Moreover, the price of these devices is low, so they may be added to the system without incurring large costs.

Considering software, there are numerous steganographic methods designed for video files. Because multimedia formats store both audio and video streams, some algorithms base on image and audio steganography [7] or various combinations of them [58]. In image steganography we may encounter solutions based on the least significant bit combined with cryptography which are designed to prevent cybercrime in hotels [59] and additionally demonstrate some resistance to steganalysis. There are also techniques which use specific features of a codec, for example, ref. [8] describes an algorithm of data hiding that modifies the motion vector of fast objects. Motion estimation is used for saving space, as in most cases adjacent frames are similar, so storing all these data would be redundant. This topic raised the interest of researchers who invented data hiding methods [60,61,62] as well as detection techniques [63,64,65]. Another branch of steganography uses artificial intelligence to achieve specific goals, like better visual quality of stego video, robustness of the carrier etc. An example of such technique is [66] in which genetic algorithm (nature-inspired iterative method with reproduction, crossover and mutation) has been used to optimize embedded pixels coefficients in LSB method. Video carriers also find application in watermarking to allow validation even if the file is damaged. An example is described in [67]—it uses a steganographic method to combine security with authentication. This approach is different from presented in this research, in which emphasis is put on information hiding.

As can be seen, the majority of video steganography algorithms are quite complex. Usually, it is not a problem, but in the presented project, one of the goals is saving resources. This is because IoT systems carry out multiple tasks, so hiding data cannot be too expensive in computational terms. For this reason, metastego and videostego—algorithms of low complexity—have been chosen. A bit limited security has been compensated by additional encryption. The second reason for these algorithms is their operation space—they work independently of each other and do not overwrite stored secrets.

A few difficulties were connected with data entry. In the touch sensor, the operator needs either to memorize Morse code nor to use a cheatsheet. In the latter case, smaller number of characters can be used as input in the same period of time, so the throughput is limited for untrained users. No similar issues were observed for the joystick, which turned out to be easier to learn. Considering both sensors, the best results were achieved for short messages. For longer data, the human factor plays a role, as users started to be tired, make occasional errors or lose focus. Other potential problems related to software (like modifications done by popular platforms) do not apply to described application.

## 5. Conclusions

The realization of multi-secret steganographic system shown in this paper accomplished the assumed objectives. The selected sensors are easy to use and do not attract too much attention. In this way, they are practical in steganographic applications. The algorithms chosen for data embedding are characterized by low complexity so that they do not distort any remaining operations in the IoT system. Additionally, they do not interfere with each other, which gives us flawless recovery of all hidden messages. The presented setup turned out to be the best compared to other tested devices and methods.

Future studies may reach new platforms and types of sensors, including wearable and implantable devices. With more efficient hardware, it would be possible to use advanced methods focused on undetectability or robustness to processing. New systems should also require invention of fitting interfaces, should be convenient for users and fault-tolerant. Another possible direction involves fortifying the system with cryptographic devices and enriching the security with fast hardware encryption. The IoT network is growing, but privacy issues are still a niche filled with numerous challenges.

## Figures and Tables

**Table 1:** Summary statistics of lengths of most frequent words in English.

Category	Min	Median	Mean	Max
Top1000	1	4	5.688	14
Top2000	1	4	6.126	14
Top3000	1	5	6.365	15

**Table 2:** Summary statistics of lengths of number of gestures needed.

Encoding	Category	Min	Median	Mean	Max
Telephone	Top1000	1	12	12.53	37
	Top2000	1	13	13.5	37
	Top3000	1	13	14.06	39
Morse	Top1000	2	14	14.72	38
	Top2000	2	15	15.75	38
	Top3000	2	15	16.36	44

## Data Availability

Data sharing not applicable.
